# Coping Styles in Patients with Anxiety and Depression

**DOI:** 10.5402/2012/128672

**Published:** 2012-06-18

**Authors:** Pashtoon Murtaza Kasi, Haider Ali Naqvi, Abaseen Khan Afghan, Talha Khawar, Farooq Hasan Khan, Umber Zaheer Khan, Urooj Bakht Khuwaja, Jawad Kiani, Hadi Mohammad Khan

**Affiliations:** ^1^Department of Psychiatry, Medical College, Aga Khan University, Karachi 74800, Pakistan; ^2^Department of Psychiatry, Aga Khan University, Karachi 74800, Pakistan

## Abstract

Different individuals use different coping styles to cope with their problems. In patients with anxiety and/or depression, these have important implications. The primary objective of our study was to estimate the frequency of different coping mechanisms used by patients with symptoms of anxiety and depression. A descriptive, cross-sectional survey was conducted and patients with symptoms of anxiety and depression were identified using the Aga Khan University's Anxiety and Depression Scale (AKUADS). Coping styles were determined by using the 28-item Brief COPE inventory. We were able to recruit 162 people. The prevalence of anxiety and depression was found to be 34%. Females were more than 2 times likely to have anxiety and depression (*P* value = 0.024, OR = 2.62). In patients screening positive for AKUADS, “religion” was the most common coping mechanism identified. “Acceptance”, “Use of instrumental support”, and “Active coping” were other commonly used coping styles. Our findings suggest that religious coping is a common behavior in patients presenting with symptoms anxiety and depression in Pakistan. Knowledge of these coping styles is important in the care of such patients, as these coping methods can be identified and to some extent modified by the treating clinician/psychiatrist.

## 1. Introduction

Different individuals use different strategies for coping with negative affective state and associated life problems [1]. Strategies are developed to identify means to reduce stress. Such coping mechanisms are important both in periods of acute stress/emergencies (such as hurricane disasters) as well as in patients suffering from chronic illnesses such as depression, breast cancer, and HIV/AIDS. 

The use of some of these coping styles may prove beneficial for the person. For example, in a study on the coping mechanisms and depression in elderly medically ill men, a high proportion of the respondents sought comfort in religious beliefs and practices. This in turn was inversely related to their severity of depression [2]. 

On the other hand, the use some of these coping styles, such as “substance use,” may be termed as “maladaptive” and may result in poorer health outcomes for the patient [3].

 In chronic diseases, such as depression and anxiety, knowledge of these coping styles by the treating clinician/psychiatrist can have important implications. We, therefore, studied the frequency of these different coping styles in patients with symptoms of anxiety and depression presenting to primary health care settings of Pakistan. 

## 2. Methods

### 2.1. Objectives 

#### 2.1.1. Primary Objective

To identify and estimate the frequency of the different coping mechanisms used by patients with symptoms of anxiety and depression. 

#### 2.1.2. Secondary Objective

To estimate the prevalence of anxiety and depression in patients attending three primary health care settings of Karachi, Pakistan.

### 2.2. Patients and Methods

A cross-sectional survey was conducted at three primary health care centers between September to October 2004. The three primary health care centers at Hijrat Colony, Sultanabad, and Rehri Goth were chosen because they represent the semiurban and rural settings of Karachi and Pakistan. Additionally easy access and complete demographic profile of the community were available because of an ongoing Urban Health Program (UHP) at these centers by our hospital. A team of seven medical students were trained in the use of questionnaire for assessing depression, anxiety, and coping strategies. Informed consent was obtained before the administration of the questionnaire.

### 2.3. Questionnaire

The questionnaire had 4 parts and collected information on basic sociodemographic characteristics, the Aga Khan University Anxiety and Depression scale (AKUADS), 28-item Brief Cope Inventory, and other questions relating to aspects such as substance abuse, self-medication, and religious practices. 

#### 2.3.1. Aga Khan University Anxiety and Depression Scale (AKUADS)

The scale is an indigenously developed screening instrument to be used in the community for the assessment of psychiatric morbidity in the population. The questionnaire comprises 25 questions, with 13 addressing psychological and 12 somatic symptoms. Each question gives 4 choices to the respondent (never: 0, sometimes: 1, mostly: 2, and always: 3) and the total score is determined by adding the scores for each of the responses [4]. At a score of 20 the instrument has a sensitivity of 66% and a specificity of 79%. The tool has been used in a variety of settings and the validity of the instrument is also more than the previously used instruments, as it includes both the psychiatric and somatic aspects of the disease, hence making it a suitable tool to be used in the community setting.

#### 2.3.2. Brief Cope-28

**Figure 1:**
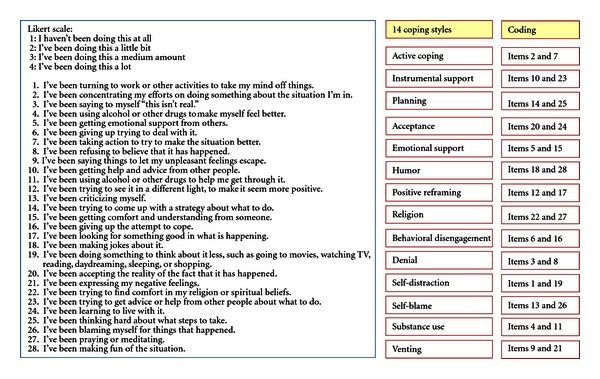
The Brief COPE tool [5] (available online: http://www.psy.miami.edu/faculty/ccarver/sclBrCOPE.html).

Coping strategies employed in the past 1 month by the participants screened as having depression and anxiety were determined with the use of Brief COPE Inventory [5]. The Brief COPE developed by Carver at the University of Miami is one of the most commonly used coping measures and has been cited by more than 900 articles as of August 2011. It is the abridged version of the original COPE Inventory and assesses 14 coping types with 28 questions (2 questions per type; see Figure 1). These include, for example, “active coping” (I have been taking action to try to make the situation better), “religion” (I have been praying or meditating), “venting” (I have been expressing my negative feelings), and “substance use” (I have been using alcohol or other drugs to make myself feel better).

Since most of the participants presenting to primary health care settings are not literate, these items were rephrased into questions so that the questionnaire became interviewer based. For example, for “substance use,” “Have you been using alcohol or other drugs to make yourself feel better?” These items were then translated into Urdu by authors P. M. Kasi and U. Z. Khan. A back translation was then made by Fahd Khalid Syed and compared with the original version. The discrepancies were then corrected. The final version was then given for approval to Naqvi Sahab (Learning Resource Center, Aga Khan University) to check the validity of the translated items in Urdu Language. 

The responses to these questions are measured on a 4-point Likert-type scale with responses ranging from 1 (“I've not done this at all”) to 4 (“I've been doing this a lot”). The scores (ranging from 2 to 8) and the means for each coping method were then calculated.

**Figure 2:**
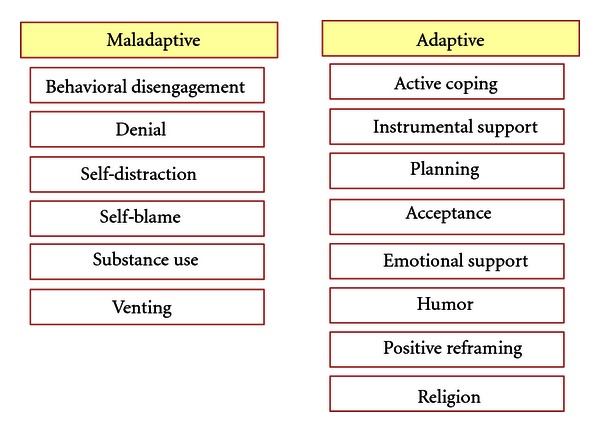
Suggested Grouping of the 14 coping scales into adaptive versus maladaptive coping strategies.

Although each of these coping strategies overall cannot be termed as adaptive or maladaptive and are dependent on the context and situation, the suggested grouping used in some of the previous studies is outlined in Figure 2. 

It is important to note that the author of the Brief COPE tool did not attempt to dichotomize the coping scales into positive or negative coping strategies, as “different samples exhibit different patterns of relations.” This task has been left to the researchers utilizing the Brief COPE tool and has been reported in various ways in the literature [5].

## 3. Results

### 3.1. Basic Sociodemographic Characteristics

These are summarized in Table 1. We were able to recruit 162 people into our study. More women report to these primary health care centers, thus their number gets higher (74.1%). The areas interviewed represent the underprivileged class of the society, where illiteracy was seen to be high (52.5%), with more than half of the families having a monthly income of less than Rs. 5,000 (*≲*$85 US). Other surrogate markers of socio-economic status, such as the “type of house” and “house ownership,” which have been used in the national health survey were also used and the results shown.

### 3.2. Anxiety and Depression

Out of 162 people interviewed, 55 individuals screened positive for anxiety and depression (AKUADS score ≥19). This translates to a prevalence of 33.95%. Females were significantly more likely to have symptoms of anxiety and depression than men (*P* value = 0.024, OR = 2.62). On the other hand, marital status, mother tongue, and other measures of socioeconomic status were not significantly associated with anxiety and depression (Table 2).

### 3.3. Coping Styles

Out of the 55 individuals who had symptoms of anxiety and depression, 3 did not fill the “cope” part of the questionnaire. Analysis of coping styles was, therefore, carried out on the results of 52 individuals.

**Figure 3:**
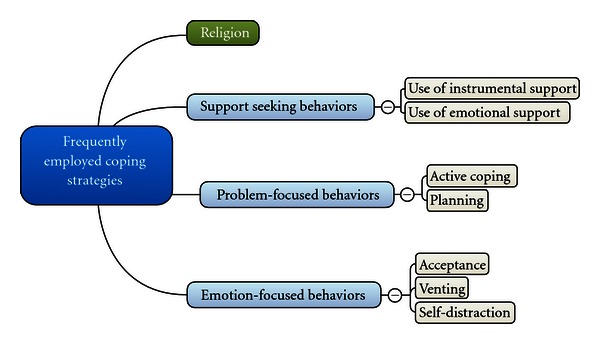
Categorization of the frequently employed coping strategies seen in patients with anxiety and depression.

Of the 14 coping styles studied, the most frequently used strategies were religion (48.1%), acceptance (34.6%), Use of instrumental support (32.7%), active coping (30.8%), planning (28.8%), and use of emotional support (28.8%), using the particular coping style on a moderate (“I do this most of the time”) to frequent (“I do this all the time”) basis (Figure 3).

The other 8 coping styles were used less frequently by individuals with symptoms of anxiety and depression, with humor (9.6%), behavioral disengagement (7.7%), and substance use (5.8%) being used least frequently (see also Table 3).

### 3.4. Type of Religious Coping

**Figure 4:**
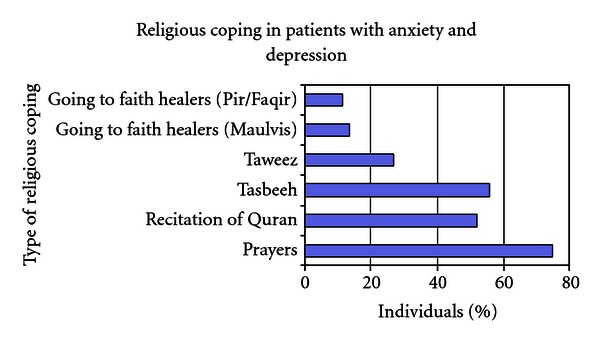
Different religious coping styles employ.

With respect to “religion” as a coping mechanism, we also inquired on the particular method used. As shown in Figure 4, 75% of the individuals with anxiety and depression found comfort or were praying to feel better, and more than half sought comfort by recitation of the Holy Book (Quran) or by frequent remembrance of God (Tasbeeh). It is also interesting to note that a quarter of the individuals had also visited a Faith Healer for their symptoms. *Taweez*, also a remedy to problems given by Faith Healers, was sought by 27% of the individuals with symptoms of anxiety and depression.

## 4. Discussion

Across the three primary health care centers, we found the prevalence of anxiety and depression to be around 34%. This is similar to studies done in similar settings in Pakistan, with the prevalence ranging between 30.4 to 38.4% [6, 7]. A recent review in BMJ on anxiety and depressive disorders in Pakistan also estimated the overall prevalence to be around 34% (29–66% for women and 10–33% for men) [8]. 

We studied the frequency of different coping styles and found *religious coping* as the most common behavior, with significant proportion of individuals finding comfort and relief in different religious practices.

Substance use as a coping mechanism was minimal. A probable explanation for lowest scores on questions of substance abuse, that is, “use of alcohol or other drugs to feel better or help the individual get through his/her symptoms,” is first, and the data represents mainly women of low socio-economic strata of Pakistan. Men are more likely to be involved in substance abuse, and alcohol in particular is more in use in the higher socioeconomic classes of Pakistan. Islam also prohibits alcohol, and all individuals, except one, in our study were Muslims. 

It is, however, interesting to note that there is much benzodiazepine abuse, where most of these drugs are available over the counter, with many individuals self-medicating. When asked about whether or not the person self-medicated to feel better, we found 30.8% have been doing so, with 19.2% of the individuals doing this on a moderate to frequent basis. This is alarming. We have not looked into the drugs they were using but a center-based study in our hospital by Khawaja et al. (personal communication) found this to be close to 30–40%, which is also consistent with our findings [9, 10]. It can, therefore, be suggested that in our context self-medication should be studied as a separate coping mechanism and the treating physician/psychiatrist should be aware if his patient is doing so.

Coping mechanisms are also associated with the patient's understanding of his disease/symptoms and the ways in which he manages his illness. For example, endorsement of depressive symptoms was found to be associated with more self-blame and emotional venting. Perceived negative consequences of depression led to more active coping, religious coping, and self-blame, whereas perception of disease as chronic led to less planning on the part of the patient [11]. Coping behaviors are thus affected by a number of parameters and being aware about your patients' coping styles to manage his depression could in turn guide you while providing him with treatment.

Similarly, “active coping” was associated with lower levels of anxiety in accident and emergency house officers. Coping by “venting,” on the other hand, produced greater levels of anxiety and depression [12].

The weaknesses of the current study include the cross-sectional design of the study and employing tools that use self-reported data to assess the severity of symptoms and the frequency of usage of a particular coping strategy. Causality thus cannot be established. Use of screening tool (AKUADS) to establish depression and further assess its severity is itself another limitation.

Currently in developing countries like Pakistan, there is dearth of mental health professionals and services. For an estimated population of more than 150 million, which is mostly rural, there are only 342 practicing psychiatrists, with most of them located in major cities [13]. Majority of patients present to general physicians and other specialists. In the light of previous studies, it may, therefore, be important to empower patients with positive coping styles or discourage negative coping styles to improve their overall quality of life, thus providing clinicians/psychiatrists with a useful tool as coping behaviors are modifiable. Further prospective studies would be needed to corroborate the effect of such an intervention [14]. 

## Figures and Tables

**Table 1:** Basic socio-demographic characteristics.

No.	Characteristic	*n*	%
1	Sex		
	Females	121	74.7
	Males	41	25.3

2	Marital Status		
	Unmarried	35	21.6
	Married	117	72.2
	Divorced	2	1.2
	Separated	2	1.2
	Widowed	6	3.7

3	Number of children		
	0	11	6.8
	1-2	38	23.5
	3-4	36	22.2
	5-6	25	15.4
	>6	16	9.9

4	Education		
	Uneducated	85	52.5
	Primary	30	18.5
	Secondary	40	24.7
	Inter/Graduate	7	4.3

5	Occupation		
	Housewife	94	58.0
	Community worker	31	19.1
	Laborer	13	8.0
	Shopkeeper	7	4.3
	Fisherman	6	3.7
	Others	11	6.8

6	Monthly income		
	<5,000	87	53.7
	5,000–10,000	56	34.6
	10,000–20,000	16	9.9
	>20,000	3	1.9

7	House ownership		
	Yes	115	71.0
	No	47	29.0

8	Type of house		
	*Kacha* (“Mud House”)	47	29.0
	*Semi-Pakka* (“Brick House”)	94	58.0
	Flat	10	6.2
	Bungalow	11	6.8

9	Mother tongue		
	Urdu	9	5.6
	Sindhi	56	34.6
	Punjabi	15	9.3
	Pashto	43	26.5
	Hindko	26	16.0
	Others	13	8.0

10	Area		
	Hijrat Colony	61	37.7
	Sultanabad	42	25.9
	Rehri Goth	59	36.4

Total		162	

**Table 2:** Anxiety and depression results.

No.	Variable	*f*	*n*	%	*χ* ^2^	*P*-value
1	Sex					
	Males	8	41	19.5	5.10	0.024
	Females	47	121	38.8		(OR = 2.62)

2	Marital status					
	Unmarried	11	35	31.4	1.16	0.559
	Married	42	117	35.9		
	Others	2	10	20.0		

3	Mother tongue					
	Urdu	4	9	44.4	2.90	0.716
	Sindhi	19	56	33.9		
	Punjabi	6	15	40.0		
	Pushto	11	43	25.6		
	Hindko	11	26	42.3		
	Others	4	13	30.8		

4	Education					
	Uneducated	31	85	36.5	0.54	0.765
	Primary	9	30	30.0		
	Secondary and higher	15	47	31.9		

5	Monthly income (Pakistani Rupees)					
	<5,000	31	87	35.6	2.92	0.233
	5,000–10,000	15	56	26.8		
	>10,000	9	19	47.4		

6	House ownership					
	Yes	39	115	33.9	0.00025	0.99
	No	16	47	34.0		

**Table 3:** Coping style scores (Brief Cope-28) in patients screening positive for anxiety and depression.

Brief COPE coping style	Mean	SD	Range
Religion	5.46	1.84	2–8
Acceptance	4.73	1.59	2–8
Use instrumental support	4.71	1.38	2–8
Use of emotional support	4.63	1.88	2–8
Self Distraction	4.56	1.41	2–8
Active coping	4.48	1.63	2–8
Planning	4.38	1.62	2–8
Venting	4.15	1.66	2–7
Self-blame	3.83	1.67	2–8
Denial	3.67	1.49	2–8
Positive reframing	3.63	1.33	2–8
Humor	3.31	1.72	2–8
Behavioral disengagement	3.31	1.46	2–8
Substance abuse	2.54	1.20	2–7

## Abbreviations

AKUADS: Aga Khan University Anxiety and Depression Scale.
